# Beta-blockers in patients with liver cirrhosis: Pragmatism or perfection?

**DOI:** 10.3389/fmed.2022.1100966

**Published:** 2023-01-09

**Authors:** Tilman Sauerbruch, Martin Hennenberg, Jonel Trebicka, Robert Schierwagen

**Affiliations:** ^1^Department of Internal Medicine I, University of Bonn, Bonn, Germany; ^2^Department of Urology, University Hospital, Ludwig Maximilian University of Munich, Munich, Germany; ^3^Department of Internal Medicine B, University of Münster, Münster, Germany; ^4^European Foundation for the Study of Chronic Liver Failure, Barcelona, Spain

**Keywords:** beta-blockers, portal hypertension, liver cirrhosis, signaling, carvedilol, adherence

## Abstract

With increasing decompensation, hyperdynamic circulatory disturbance occurs in liver cirrhosis despite activation of vasoconstrictors. Here, the concept of a therapy with non-selective beta-blockers was established decades ago. They lower elevated portal pressure, protect against variceal hemorrhage, and may also have pleiotropic immunomodulatory effects. Recently, the beneficial effect of carvedilol, which blocks alpha and beta receptors, has been highlighted. Carvedilol leads to “biased-signaling” *via* recruitment of beta-arrestin. This effect and its consequences have not been sufficiently investigated in patients with liver cirrhosis. Also, a number of questions remain open regarding the expression of beta-receptors and its intracellular signaling and the respective consequences in the intra- and extrahepatic tissue compartments. Despite the undisputed role of non-selective beta-blockers in the treatment of liver cirrhosis, we still can improve the knowledge as to when and how beta-blockers should be used in which patients.

## Prologue

In liver cirrhosis there is a significant change in hemodynamics in different vascular compartments, depending on the degree of decompensation (1–3). This is a major cause of organ dysfunction, concerning not only the liver but also the kidney (4), the lungs (5), the heart (6) or the intestine (7). Regardless of the etiology of cirrhosis, vasodilatation of the splanchnic vessels occurs early, followed by peripheral vasodilatation with decreased intrathoracic blood volume, resulting in hormonal counter regulation and hyperdynamic circulatory disturbance (2, 8, 9). Within the liver–in addition to the remodeling of the organ architecture and contrary to the extrahepatic situation–vasoconstriction dominates (2).

One force driving toward decompensation of liver cirrhosis is portal hypertension, often alongside a chronic inflammatory status. Such inflammation is caused and maintained on the one hand by etiological factors such as viruses, which directly damage the liver, and on the other hand by potential stimuli derived from the bowel. Based on these pathophysiological mechanisms, the treatment of liver cirrhosis primarily aims at the interruption of etiology and in advanced stages of liver disease additionally at reduction of portal hypertension and its sequels–a main cause of complications–either by drugs or placement of a *trans*-jugular intrahepatic portosystemic shunt (TIPS).

In this context, administration of a non-selective ß-blocker (NSBB) has been firmly established for almost four decades. The therapy was introduced by Lebrec and coworkers under the hypothesis that a non-selective ß-blocker reduces tributary blood flow into the portal vein (10), thereby diminishing the risk of bleeding from varices. This hypothesis passed the test of a clinical trial followed by many others (11). In Germany, the admission rate for variceal bleeding decreased significantly between 2005 and 2018, possibly due to the widespread use of ß-blockers in liver cirrhosis (12).

Non-selective ß-blockers have been used for primary and secondary prophylaxis of variceal hemorrhage (13, 14), and there is much evidence that NSBBs reduce the risk of first and recurrent bleeding from esophageal varices. Less certain is to which degree and whether this also has an effect on survival. Furthermore, there is still uncertainty as to which patients respond to the administration of a NSBB. Also, there is now a body of evidence pointing to beneficial pleiotropic effects of NSBBs beyond their effect of lowering blood flow and blood pressure in the portal vein and its collaterals.

## Hemodynamic changes in liver cirrhosis, catecholamines, their respective receptors and signaling

More than 60 years ago, it was observed that patients with liver cirrhosis have hyperdynamic circulation disorder, characterized by an increased cardiac index (CI) and a decreased systemic peripheral resistance (15–17). This disturbance increases with the extent of decompensation of liver cirrhosis. It is less dependent on the etiology. Especially in the abdomen, vasodilation occurs early as a result of portal hypertension, causing a shift of blood from the intrathoracic compartment into the splanchnic vasculature. Mediators for this phenomenon are vasodilators that act systemically and paracrine, especially nitric oxide (NO), but also other molecules such as carbon monoxide (CO), prostacyclines (PGl_2_) or glucagon (1, 16, 18). One stimulus for formation of these molecules is believed to be vascular shear stress (19) in the splanchnic area (especially at onset of portal hypertension). Another is a subclinical chronic inflammation, of which it is increasingly discussed that a disturbed intestinal barrier and translation of pathogen-associated molecular patterns (PAMPs) from the gut into the body are the cause, together with an intestinal dysbiosis (20). The inhibitory effect of certain bile acids on vascular smooth muscle cell (VSMC) contraction may also play a role (21). The process is additionally maintained by an impaired VSMC response to vasoconstrictors, especially in decompensated cirrhosis (1, 22–24).

### Adrenergic stimulation in liver cirrhosis

Around 40 years ago, the research group of Robert Schrier showed that plasma norepinephrine (NE) levels are significantly elevated in patients with decompensated liver cirrhosis as compared to controls (25). This is associated with water retention. By elegant investigations (head-out water immersion) they could show that it is mainly a reaction to a reduced arterial blood volume, i.e., vascular underfilling where intrathoracic baroreceptors react. The high plasma NE levels correlate significantly with vasopressin levels (26) and in cirrhosis with ascites they are associated with high plasma renin and aldosterone levels, as a consequence of an activated renin-angiotensin-aldosterone-system. Furthermore, high plasma vasoconstrictors correlate positively with the degree of portal hypertension (27). These plasma levels reflect a sympathetic overactivity induced by baroreceptor-stimulation, but may be also due to an overflow of local organ NE formation, such as in the kidney, liver, and heart. Furthermore, the central nervous system is involved. The contribution of the different organ compartments to the systematic plasma concentrations is difficult to differentiate. However, the activation of the baroreceptors is an essential source (28).

### Adrenergic receptors

Catecholamines like norepinephrine and epinephrine, which–as stated above–increase with decreased liver function in cirrhosis, mediate their effect *via* adrenergic receptors which are G protein-coupled (GPCR). The effect of the sympathetic system depends on the expression of different receptors on the various cells and organs. They are categorized into two main groups: α and β receptors with nine subtypes (α_1_ and α_2_, with three subtypes each, as well as the ß_1_, ß_2_, and ß_3_ receptors). All three ß-adrenergic receptors (ß-AR) are coupled on their cytoplasmic side to G_*s*_ proteins, and in the case of ß_2_- and ß_3_-AR also to G_i_ proteins. Comparing the potency of norepinephrine and epinephrine, epinephrine has a stronger effect on ß_2_- and norepinephrine has a stronger effect on ß_1_-AR. Both have about the same effect on α_1_ receptors (29–31).

Although cardiac myocytes predominantly express ß_1_-AR and peripheral vasculature or the bronchial system predominantly express ß_2_-AR, most organs have a heterogeneous composition of ß-receptors. For instance, ß_1_-AR of the kidney are involved in renin secretion, whereas ß_2_-AR directly influence sodium concentration in the tubular system *via* ion channels/transporters (32). ß_3_-AR are expressed in a variety of human tissues such as skeletal muscle, atrium, heart, adipose tissue, brain or–to a great extent–in the urinary bladder (33–35). We found an up-regulated expression of ß_3_-AR in arteries and the liver in the condition of liver cirrhosis (36).

Thus, with respect to the cardiovascular and intestinal systems, activation of ß_1_-AR causes an increase in the frequency and contraction of the heart, whereas activation of ß_2_-AR causes vasodilation. The motility of the intestine and gallbladder is decreased by ß_2_-agonists.

While we know that the sympathetic system is activated in cirrhosis, we have little insight beyond systemic levels into the extent of activation at the cellular and organ levels across stages of cirrhosis. Even less is known about how the expression of the various adrenergic receptors change at the cellular level in the different organs during liver cirrhosis.

### Signaling and ß-receptors

The contraction and relaxation of smooth muscle cells depends largely on the phosphorylation and dephosphorylation of myosin light chains (MLC), essential contractile proteins. Here, calcium homeostasis, regulated by its *trans*-membrane influx against efflux of potassium plays an important role. Thus, vascular smooth muscle contraction by activation of vasoconstrictor receptors, including α_1_-AR or receptors for angiotensin-II result in contraction by promotion of MLC phosphorylation caused by three prototypical intracellular signaling pathways, shared by all contractile receptors (1).

**FIGURE 1:**
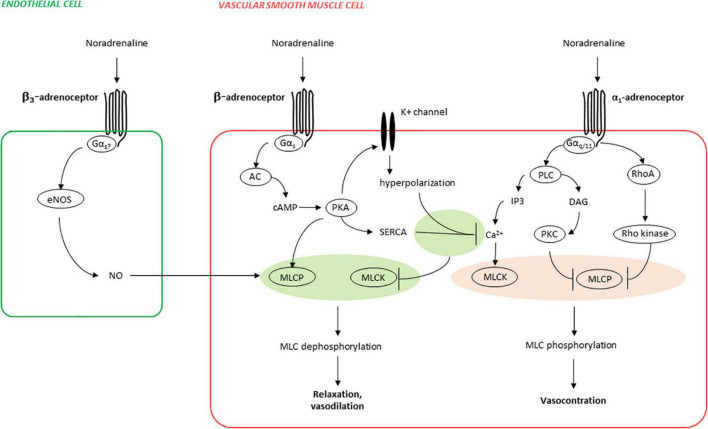
Mechanisms of ß-adrenergic vasorelaxation and α_1_-adrenergic vasocontraction (simplified). Phosphorylation of myosin light chains (MLC) in smooth muscle cells is essential for contraction and is increased by MLC kinase (MLCK) and decreased by MLC phosphatase (MLCP), both being adversely regulated by α_1_- and ß-adrenoceptors. Activation of α_1_-adrenoceptors causes activation of phospholipase C (PLC), resulting in inositol-1,4,5-trisphosphate (IP3) formation, leading to increases in cytosolic calcium concentrations and finally to contraction by calcium-dependent MLCK activation. In parallel, MLC phosphorylation is promoted by deactivation of MLCP, caused by its inactivation by protein kinase C (PKC) activation by PLC-derived diacylglycerol (DAG), and in parallel by RhoA/Rho kinase. Activation of ß-adrenoceptors (predominantly ß_2_-AR) causes activation of adenylyl cyclase (AC), subsequent cyclic adenosine monophosphate (cAMP) formation, and activation of the cAMP-dependent protein kinase A (PKA). PKA activates several potassium channels, resulting in hyperpolarization and finally in decreases of cytosolic calcium concentrations by closure of voltage-dependent calcium channels. In parallel, PKA-mediated decreases in cytosolic calcium are imparted by relocation of calcium from the cytosol to intracellular stores, resulting from PKA-mediated sarco/endoplasmatic Ca^2+^-ATPase (SERCA) activation. Finally, PKA activates MLCP, leading to reduced MLC phosphorylation and relaxation, in addition to PKA-mediated decreases in cytosolic calcium. In endothelial cells, ß_3_-adrenoceptors may activate endothelial nitric oxide synthase (eNOS), resulting in production of the vasodilator nitric oxide (NO), and NO-mediated vasorelaxation.

Vascular ß-AR are coupled to G protein alpha subunit (Gαs) (see Figure 1; 37). Activation of Gαs by ß-adrenergic receptors causes intracellular adenylyl cyclase activation, subsequent cAMP production, and cAMP-dependent relaxation in smooth muscle cells or contraction in cardiomyocytes, by various mechanisms (34, 37). One major mechanism in smooth muscle cells is activation of protein kinase A (PKA) by cAMP, resulting in relaxation by decreasing cytosolic calcium concentrations and calcium sensitivity (see Figure 1; 38, 39). ß_3_-AR mediate their effects probably mainly *via* Gαs in smooth muscle cells, but also *via* Gi and eNOS, at least in endothelial cells and cardiomyocytes (34). The latter leads to formation of cGMP and vasodilatation (34). Their vasodilatory effect may also be further induced *via* inhibition of Rho-kinase (36).

However, a number of other responses of smooth muscle cells may occur after activation of ß-AR by recruiting other G-proteins or non-G protein interaction partners, altering the membrane localization of the receptor. As a result, other intracellular signaling proteins are activated (40). Here, the phenomenon of ß-arrestin recruitment to activated GPCR is of clinical relevance with a transduction of signaling toward other pathways (41)–besides the receptor desensitization effect of ß-arrestin. Principles of this phenomenon have been described, especially for activation of ß_2_-AR and angiotensin II receptors (1, 42). Evidence that ß-arrestin-mediated receptor regulation also applies to splanchnic vessels in liver cirrhosis is available, at least for angiotensin II receptors (24, 43, 44).

## The different ß-AR blockers

About 60 years ago, ß-blockers were introduced for the treatment of systemic hypertension (45). Although all ß-blockers have an antihypertensive effect they differ in pharmacokinetics and pharmacodynamics, depending on their molecular structure (29). ß-AR blockers can be broadly divided into water-soluble and lipid-soluble agents, as well as into ß_1_ selective and non-selective substances.

At high concentrations, the selective ß_1_-effect is partially lost. Some ß-blockers have an additional agonistic effect on the ß-AR (intrinsic sympathomimetic activity, e.g., pindolol), whereas carvedilol has an additional antagonistic effect on α-receptors. Nebivolol also promotes NO formation (39; Table 1). Conventional NSBB or ß_1_-AR blockers have only a minimal effect on ß_3_-AR (34).

**TABLE 1:** Non-selective ß-blockers used for therapy of portal hypertension.

Drug	ß_1_–blockade potency ratio (propranolol = 1) (29)	Further effects	Daily oral dosage^#^
Carvedilol	10	α_1_ blocking activity, biased signaling *via* ß-arrestin	Start with 1 × 6.25 mg (even lower) up to 2 × 6.25 mg
Nadolol	1.0		1 × 20–40 mg up to 1 × 160 mg
Propranolol	1.0		2 × 20 mg up to 2 × 80 mg
Timolol	0.6		1 × 10 mg up to 1 × 80 mg

^#^Resting heart rate 55–70 beats/min, systolic BP > 90 mm Hg.

Propranolol and metoprolol are lipophilic. They are almost completely absorbed *via* the intestine and largely metabolized by the liver. Thus, their bioavailability is quite variable. They also have a short plasma half-life. Nevertheless, mainly due to the receptor binding, a dosage twice daily or–in other galenic forms–even once daily is sufficient.

Most of the trials in patients with liver cirrhosis have been performed with non-selective ß-blockers (NSBBs) propranolol, nadolol, timolol or carvedilol (Table 1).

**FIGURE 2:**
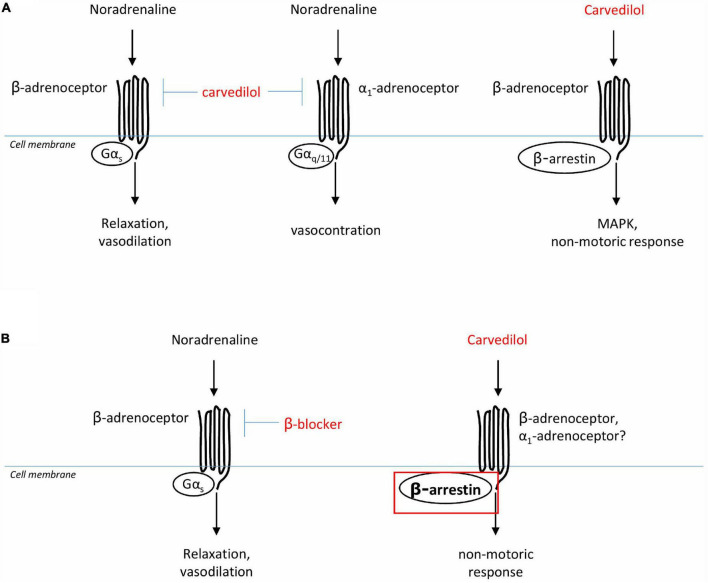
Presumed actions of ß-AR blockers on vascular smooth muscle cells. **(A)** Carvedilol is an adrenergic ligand, antagonizing ß- and α_1_-adrenoceptors, and also inducing biased signaling by ß-arrestin. **(B)** In portal hypertension, ß-blockers are presumed to reduce splanchnic vasodilatation, and therewith portal tributary blood flow and portal pressure, by antagonism of ß-adrenoceptors. It could be speculated that in liver cirrhosis the biased signaling effect of carvedilol is even more pronounced due to up-regulation ß-arrestin in these patients.

Among the different ß-blockers, carvedilol shows a unique pharmacological profile, which is, as mentioned above, first reflected by antagonism of α_1_-AR in addition to blockade of ß-AR (Figure 2). Antagonism of α_1_-AR by ß-adrenergic ligands is not uncommon, but often requires unphysiologically high concentrations (46, 47). However, binding of carvedilol to ß- and α_1_-AR occurs with similar affinities, and within ranges of plasma concentrations (see Figure 2; 48, 49). Plasma levels with daily doses of 12.5 and 25 mg range from 115 to 131 nM in healthy patients, and from 256 to 315 nM in patients with chronic renal insufficiency (50). Thus, antagonism of α_1_-adrenoceptors by carvedilol occurs *in vivo*, especially in kidney dysfunction, even though its clinical relevance has been debated (37). In contrast to most other NSBBs, carvedilol may not only “block” ß-adrenergic receptors, but may even activate ß-arrestin-induced signaling, a behavior allowing classification as a “biased ligand” (37, 51). Functional outcomes of signaling *via* ß-arrestins are unknown in the context of cirrhosis and with respect to portal hypertension, splanchnic vascular cells or the heart in liver dysfunction. ß-arrestin dependent pathways include non-motoric functions, such as activation of mitogen-activated protein kinases (MAPK) with consequences on proliferation, differentiation, and growth of cells (37). See below!

It is evident that NSBBs lower cardiac index *via* ß1 blockade. But it is poorly studied what effect they have at the different vessel compartments in liver cirrhosis.

## Beta-arrestin, biased signaling, and carvedilol

ß-arrestin-1 and -2 are ubiquitously expressed intracellular proteins that modulate the response to stimulation of GPCRs (52). By binding to phosphorylated GPCRs, they desensitize G-protein mediated signaling in the cell, partially through receptor endocytosis (53). In addition to this canonical role of ß-arrestin, current attention is increasingly focused on its function as a scaffold protein, which–internalized with other intracellular proteins–triggers further pathways as a cytoplasmic “signalosome”, or directly by receptor GPCR binding, and thus inducing different patterns of signaling cascades in the cell. This phenomenon has been described particularly for activation of the angiotensin II receptor, but most likely also applies to other adrenergic receptors (1, 52, 54).

Increased ß-arrestin signaling has been associated with profibrotic diseases (55, 56). In liver cirrhosis, increased expression of ß-arrestin 2 was found in liver (57), gastric mucosa (58, 59), and splanchnic vessels (24)–both in humans and animal models. Contrary to this, shortage of ß-arrestin 2 in sinus endothelial cells (SEC) has been described for liver cirrhosis. This may be one explanation for hepatic deficiency of NO together with increased intrahepatic resistance in liver cirrhosis, since ß-arrestin 2 mediates NO formation in SEC (60). As concerns those findings, which describe a significantly increased ß-arrestin 2 in different whole tissue homogenates in liver cirrhosis (24, 57, 58), we lack the exact assignment of which cells are involved and what increased expression of ß-arrestin in these cells causes functionally. Overexpression of ß-arrestin in splanchnic vessels in liver cirrhosis has been implicated as an explanation for the impaired vascular response to vasoconstriction (24).

Interestingly, it was then shown that patients, who respond hemodynamically to acute administration of an NSBB, express increased ß-arrestin in the stomach antrum mucosa (58, 61) and also have higher ß-arrestin serum levels (61). All these results are difficult to interpret in the absence of cell-level findings.

In the context of liver cirrhosis and alteration of ß-arrestin expression, it is interesting that carvedilol–in contrast to other NSBB–shows so-called biased signaling as mentioned above (62, 63). That is, in addition to the inhibitory effect on the G protein-dependent pathway, carvedilol induces an increased recruitment of ß-arrestin 2 by changing the conformation of the cytoplasmic portion of the receptor (64) and subsequent induction of signaling *via* ß-arrestin (51). Biased signaling has been associated with a cardioprotective effect in the cardiology literature (54, 65). As to liver cirrhosis, it would be very interesting to dissect how this alternative effect of carvedilol plays out on the cardiovascular system and other organs where an increased expression of this protein is already present.

According to the above findings, it could well be that ß-arrestin expression and binding to the ß-AR receptor (independent of carvedilol administration) increases with decompensation of liver disease, at least in the vasculature. This implies that carvedilol then acts differently with respect to a “biased-signaling” compared to individuals without liver disease (Figure 2). Thus, the functional effects of biased signaling induced by carvedilol are completely unclear in patients with liver cirrhosis to date.

ß-Arrestin is also thought to play a cancerogenic role, e.g., through a signal switch toward the wnt/beta-catenin pathway. However, despite the induction of ß-arrestin signaling, carvedilol may be protective against carcinogenesis (52, 66) at least in skin cancers (67). In animal experiments it inhibited nuclear translocation of ERK despite its effect on ERK phosphorylation (68). The effect of NSBB on HCC development is under debate (69, 70). Here, it would be interesting to have more specific data for carvedilol.

In summary, we need more information on carvedilol and its biased signaling effect in liver cirrhosis.

## Non selective ß-AR blockers and their influence on pathomechanisms of liver cirrhosis

Two main pathogenic mechanisms are relevant for deterioration and acute decompensation of patients with liver cirrhosis, namely portal hypertension and systemic inflammation (71). There is evidence that gut-barrier dysfunction and changes in the microbiome with subsequent bacterial translocation also contribute to the latter (72). In the following, we address in more detail how NSBB might influence this pathomechanism.

### The hemodynamic action of NSBB and portal hypertension

In their first study, published in the Lancet in 1980 (10), Lebrec and coworkers applied oral propranolol to eight patients with liver cirrhosis for 1 month at a dose that reduced heart rate by 25% (40–180 mg twice a day). Since then, this target reduction in heart rate has been used to dose propranolol in many centers. They showed that in all treated patients the gradient between blocked and free hepatic vein pressure (hepatic venous pressure gradient, HVPG), which–at least in alcoholic cirrhosis–correlates very well with portal pressure (73), decreased permanently. At the same time, cardiac index and hepatic blood flow (assessed by ICG method) decreased. From this, they concluded that propranolol acts *via* a reduction of splanchnic blood flow into the portal vein. None of these parameters changed in patients receiving placebo. Later they showed that there was poor correlation between the reduction in cardiac index (CI) and the decrease in HVPG. Therefore, a direct additional effect on the vessels in the splanchnic compartment was assumed. They postulated a vasoconstriction due “to unopposed alpha-adrenergic activity” as one of the factors reducing portal pressure (74).

However, a blockade of the vasodilatory effect mediated *via* the ß_2_-AR in the splanchnic arteries could also play a role.

In Lebrec’s first publication, the HVPG was reduced by 25% on average. Many other studies confirmed the portal pressure lowering effect of propranolol and other NSBB, but to a lesser extent. In the placebo-controlled studies (acute and chronic administration), the inhibitory hemodynamic effect of propranolol caused a reduction of HVPG from baseline between 10% and 22%, with a mean of about 15% (75–78). Carvedilol with its concomitant α_1_-AR blocking effect decreased HVPG by a mean of 19% (79). For propranolol, there is only a loose correlation between dose or pulse reduction and relative reduction in HVPG (76, 78, 80). For example, we found a similar reduction of HVPG and response rate (HVPG reduction of at least 20% or below 12 mmHg) in two different placebo controlled trials with either a fixed dose of propranolol (20 mg b.i.d) or a dose aimed at reducing basal heart rate by 25% resulting in total daily dosages well beyond 100 mg/day (76, 81). These findings support more recent observations that low-dose administration of NSBB may be more beneficial than high-dose administration according to the previous standard (pulse reduction by 25%) (82). A dose dependency is more likely to be found for carvedilol (78, 83).

It has been shown that for an efficient reduction of the risk of bleeding from esophageal varices, certain thresholds (>20% HVPG reduction or an absolute reduction of HVPG to below 12 mm Hg) should be reached (see paragraph surrogate marker below). Some studies demonstrated that a relative reduction of the HVPG by >10% might have a beneficial effect on survival. But it remains to be seen whether such differences can be measured reproducibly and reliably in all centers. An adequate drop in HVPG is more common in patients with high portal pressures and hyperdynamic circulatory dysfunction as compared to patients with subclinical portal hypertension–at least in compensated cirrhosis (15). However, the correlation is not strong. A later study of the group found a more pronounced effect of NSBB on systemic hemodynamics (mainly cardiac index) in patients with decompensated cirrhosis than in compensated cirrhosis, while the portal pressure decrease was smaller in these latter patients (84).

There are a number of possible explanations for the poor correlation (85) between peripheral hemodynamic criteria (CI, systemic blood pressure, pulse), the propranolol dose and the decrease in portal pressure measured by HVPG: variation in portosystemic shunts, different expression of ß-AR at the different end organs and/or different formation of vasoconstrictors in the respective patients with liver cirrhosis. In this context, it is interesting, that we found no correlation between portal venous blood flow and baseline HVPG in patients with liver cirrhosis (86). We also found that the contraction response of arteria hepatica and the portal vein of patients with liver cirrhosis to α_1_-adrenergic stimulation is impaired compared with controls. While ß_2_-stimulation showed differential effects, decreased relaxation of arteries, but increased relaxation of the portal vein in liver cirrhosis (22). One may speculate, that the ß_2_-blocking effect of NSBBs acts differently in different vascular regions. In addition, it is unclear how other NO-forming stimuli modulate the effect of NSBBs in liver cirrhosis. NO-formation increases with Child-stage (87). To complicate matters further, there is no sound knowledge as to what extent hepatic dysfunction and concomitant chronic inflammation influence expression of adrenergic receptors, cellular signaling, and phenotype, as well as plasticity of their target cells. Due to the high catecholamine levels in liver cirrhosis, down-regulation of adrenoceptors has been discussed. On the other hand, we found an up-regulation of vasopressor receptors on the transcriptional (mRNA) level in human cirrhotic hepatic arteries as compared to controls (88). Also, protein expression of all three ß-AR was up-regulated in splanchnic arteries in cirrhotic animal models and also in human arteries (ß_3_-AR) of patients with liver cirrhosis. Within the liver ß_3_-AR (humans and rats) as well as ß_2_-AR (rats) showed an increased protein expression in liver cirrhosis, but not ß_1_-AR (36). However, we do not have sufficient information about the expression of these receptors on the different cell types, their membrane localization or the induction of the respective signaling cascades.

Furthermore, different hepatic drug metabolisms, drug interactions or change of protein binding (e.g., to albumin) of the drug have to be considered. Last but not least, the reproducibility of the HVPG determination, especially in less experienced centers, should also be taken into account when interpreting results of NSBBs on hemodynamics. Sex and etiology appear to be of minor importance for the effect of NSBB in liver cirrhosis (89).

Interestingly, propranolol keeps its portal pressure lowering effect after TIPS insertion (90). Addition of irbesartan to propranolol did not further reduce HVPG, but improved natriuresis (81). Adding statins may augment the HVPG lowering effect of NSBB (91, 92), although this was debated for carvedilol (93). Concomitant phosphodiesterase-5-inhibitors further reduce portal pressure and may improve erectile dysfunction at the same time (94).

### NSBBs and inflammation

There is strong evidence in the cardiovascular literature that activation and recruitment of inflammatory cells is mediated by the adrenergic system, especially *via* ß_2_-receptors (95), and that antihypertensive drugs (96) have a beneficial immunomodulatory effect.

The hemodynamic changes that occur with increasing decompensation in liver cirrhosis are accompanied by an activation of inflammatory cells in the sense of a chronic inflammatory syndrome with concomitant immune dysfunction (3, 97, 98). A translocation of microorganisms and/or associated molecules (PAMPs) from the intestine are blamed, besides stimuli from damaged tissue (DAMPs) (99). *Via* the activation of toll-like receptors (TLR) and the inflammasome, there is a release of cytokines (100). Direct and indirect evidence suggests that NSBB can favorably influence this situation. Their administration is associated with an improvement of the intestinal barrier, reduction of bacterial translocation and activation of the immune system as measured by plasma Il-6 levels (101). Furthermore, reduction of inflammatory biomarkers in case of NSBB therapy was associated with a longer survival. This, interestingly, showed only a loose non-significant association with HVPG drop (102). Also, the incidence of spontaneous bacterial peritonitis (SBP) decreases under NSBB (103) and NSBB favorably affect at least the short-term prognosis of patients with acute on chronic liver failure (ACLF) (104). This may be explained by effects on the intestinal motility (105), but also by a direct receptor-mediated effect on immune cell signaling (95, 106–109). On the other hand, at least in inflammatory models, ß_2_-blockade has a proinflammatory effect on the kidney (32, 110). Thus, we still not fully understand how, where and when NSBBs favorably or possibly even unfavorably modulate inflammation in liver cirrhosis.

Regarding the interaction of chronic inflammatory syndrome and administration of NSBBs, it is completely unclear to what extent inflammatory stimuli in liver cirrhosis influence the expression and function of ß-AR in different organs. Corresponding changes would of course have implications for the pharmacological effect of NSBB.

### NSBBs, gut motility, and inflammation

*In vitro* studies on human colonic muscle strips could show that ß-AR are functionally expressed at the colon and that ß_1_- and (less) ß_2_-agonists lead to intestinal dilatation (111). In healthy subjects, ß-adrenergic agonists (isoprenaline as ß_2_-agonist) delayed orocoecal transit and ß-blockers (ß_1_-blocker and propranolol) accelerated it (112). Thus, one might conclude that activation of the adrenergic system in liver cirrhosis promotes constipation. The authors are not aware of adequate clinical studies on the question as to what extent intestinal motility changes with increasing decompensation of liver cirrhosis. Such trials are presumably difficult to perform due to so many other influencing factors. Nevertheless, there is some evidence of impaired intestinal motility in liver cirrhosis (113). This provides the rationale for administration of NSBBs to alter intestinal dysmotility, presumably induced by adrenergic stimulation, with the aim to influence the bacterial translocation. On the other hand, there is increasing evidence that resident macrophages in the gut favorably influence inflammation *via* ß_2_-mediated signaling (114, 115) and we do not know how intestinal dysbiosis, which has been demonstrated especially in alcoholic cirrhosis (116), affects neuroinflammatory processes in the intestine and whether NSBBs have a favorable or unfavorable effect on this.

## Indications for NSBBs, results from randomized trials

After the use of NSBBs for prophylaxis of first variceal hemorrhage and prevention of recurrent hemorrhage was established, further studies were conducted on the administration of NSBB to prevent further decompensation of compensated cirrhosis. In the following, we review the studies on these three indications (prevention of decompensation, as well as prevention of first and recurrent hemorrhage) in terms of (a) hemorrhage and (b) survival.

### Prevention of cirrhosis decompensation by NSBBs

It has long been an unanswered question whether starting the administration of NSSBs early in the course of liver cirrhosis can prevent later decompensation. Several years ago, Groszman and coworkers initiated a controlled trial to test the hypothesis, that application of NSBB in an early stage of liver cirrhosis (213 pts, over 90% Child A, around 60% hepatitis C, one fourth alcoholics, all patients without gastroesophageal varices) might prevent development of varices, bleeding or ascites, i.e., whether NSBBs can prevent signs of further decompensation (117). Timolol, a NSBB, was used. The average baseline HVPG was around 12 mmHg. Timolol dropped the heart rate by 17% and also HVPG, but non-significantly as compared to placebo. With respect to the primary endpoint (development of esophageal varices or hemorrhage from the collaterals) there was no distinction between groups (39% vs. 40%) during a median follow-up of more than 50 months. Furthermore, development of ascites and or encephalopathy (around 12% each) did not differ, nor did death rate (9% timolol group vs. 14% placebo).

*Post-hoc* analysis showed that patients with baseline HVPG < 10 mmHg, a decrease of HVPG > 10% or an increase < 10% developed significantly fewer primary endpoints irrespective of the trial group. Following these findings some centers consider a 10% drop (not 20%) in HVPG as an important prognostic threshold. See below!

In 2019, Spanish working groups again addressed the question of the extent to which NSBBs can prevent the decompensation of liver cirrhosis (118). 201 patients (one third of screened) were randomly assigned to NSBBs (propranolol, carvedilol) or placebo. Contrary to the first trial, only patients (somewhat more than 60% hepatitis C) with an HVPG > 10 mmHg were included. Response to standardized intravenous propranolol was tested at inclusion. Patients who did not show a drop in HVPG of >10% received carvedilol. The target dose was based on the reduction in heart rate (mean daily dose 95 mg for propranolol and 19 mg for carvedilol). The drop in HVPG from baseline at 1 year was higher with carvedilol (16%) than with propranolol (10%), although carvedilol was only given to iv propranolol non-responders. After a median follow-up of 37 months the composite endpoint (death, ascites, bleeding or overt encephalopathy) occurred in 16% of the ß-blocker group and 27% of the placebo group, mainly due to a reduction of ascites formation. The occurrence of decompensation correlated with a lack of drop in HVPG after 1 year. It is not entirely clear to what extent antiviral therapy for HCV-associated cirrhosis affected the outcome in the final stage of the trial, and overall there is little knowledge about the comedication in the groups. In the NSBB group 8% died, as did 11% of the placebo patients.

The results of these two studies suggest that patients with significant portal hypertension (HVPG > 10 mmHg) but not yet decompensated cirrhosis benefit–mainly regarding the formation of ascites–from treatment with NSBB, preferably carvedilol, provided there is a decrease of HVPG > 10%. It remains to be seen whether this applies to alcoholic cirrhosis, which is the major cause of cirrhosis, at least in Western countries.

### Prevention of esophageal bleeding

#### First bleeding

One main indication for application of NSBB is prevention of first bleeding from varices. In the largest placebo-controlled trial (meta-analysis of individual data from 589 patients) it has been shown that the two-year bleeding rate is reduced from 35% (controls) to 22% in the propranolol/nadolol group (119). Thus, the number of patients needed to treat (NNT) is around eight to prevent one bleeding event.

According to a recently published Cochrane review (network meta-analysis of 60 controlled trials, 6,212 patients using NSBB, nitrates, sclerotherapy or ligation of varices) on primary prophylaxis of variceal bleeding in liver cirrhosis patients with varices, NSBBs significantly reduced occurrence of any variceal bleeding compared to no active intervention in patients with varices. NSBBs or ligation (9 RCTs) were almost equal. An additional positive effect was observed by adding ligation of varices (only one trial), but there were more serious adverse events in the banding groups as compared to monotherapy with NSBB. The evidence for these findings are classified as uncertain, mainly because of low number of cases in the individual trials and the quality of the studies (120).

A very recent time to event analysis with individual patient data from 11 RCTs comprising 1,400 patients found no difference in the first bleeding rate between NSBBs (with or without ligation) and ligation only, but a lower risk to develop ascites in patients receiving NSBBs (121).

### Rebleeding

Since the first RCT (11) demonstrating the potential of NSBBs to prevent rebleeding from esophageal varices, NSBBs have become an inherent part for prophylaxis of rebleeding. Compared to placebo they reduce the rebleeding risk from 60–70% to 30–40% and combined with ligation to 25% (122). This is less than shunt procedures (5–10% rebleeding after TIPS), but does not carry the risk to augment or induce hepatic encephalopathy, an adverse event consistently shown after TIPS placement, at least in the elective situation (76, 123).

Non selective ß-AR blockers and ligation carry not only the disadvantage of a higher risk of further hemorrhage as compared to TIPS, but also the drawback not to influence the pathophysiology of salt and water retention leading to ascites, whereas TIPS–by shifting blood into the central compartment–decreases renin-angiotensin-aldosterone system (RAAS) activation and kidney salt reabsorption (124). At least one fifth of patients receiving NSBBs and ligation for prevention of rebleeding in the end was transferred to TIPS for refractory ascites (76, 123). In a recent meta-analysis, using individual patient data analysis in nearly 4,000 patients, TIPS proved superior to standard of care for rebleeding–which is in most patients ligation and NSBBs–and also with respect to further decompensation and even survival, the latter mainly due to the cohorts receiving pre-emptive TIPS after a variceal bleeding episode (125).

Most patients, who fail to respond adequately with HVPG to the administration of propranolol, respond to carvedilol. By this–compared to ligation–the primary bleeding rate can be significantly reduced (77). However, a Cochrane review (126) analyzed the randomized trials (10 RCT, 810 patients) comparing carvedilol with conventional NSBBs and found no difference with regard to primary and secondary bleeding rates and side effects, while a very recent meta-analysis (127) based on individual data showed improved survival compared to controls (ligation or placebo).

In summary, the combination of a NSBB along with ligation of varices remains the standard therapy to prevent recurrent variceal bleeding (128), although there is debate as to whether narrow-lumen TIPS, used directly in conjunction with the acute bleeding event might be the optimal recurrent bleeding prophylaxis independent from degree of decompensation and severity of bleeding, at least in patients beyond seven Child-Pugh points, mainly to protect patients from early rebleeding, which exerts an increased risk for death (129).

### Effect of NSBBs on survival of patients with liver cirrhosis

In several more recent studies, bleeding has been found to be only a minor contributor to mortality. This may explain the rather low impact of NSBBs on mortality, despite their unquestioned beneficial effect on bleeding risk. Nevertheless, the question of other pleiotropic effects of NSBBs (besides the influence on variceal hemorrhage) arises, especially for those studies that show a prolongation of survival under NSBBs as compared to alternative approaches (see above).

### Prevention of cirrhosis decompensation

Both studies investigating the effect of NSBB on decompensation of liver cirrhosis found no effect of NSBB on overall survival compared with the placebo group (117, 118).

### Prevention of first bleeding

In the first meta-analysis (119) of individual patient data (589 pts from 4 RCT) 2-year-survival was similar (68% placebo vs. 71% NSBBs). A significant beneficial effect on survival in the setting of primary bleeding prophylaxis as compared to no active intervention was described in a very recent network-meta-analysis on 6,653 patients, however with only marginal differences when NSBBs were compared to variceal ligation with and without additional NSBBs (120). A competing risk meta-analysis of 11 RCT trials comprising individual data of 1,400 patients in the setting of prevention of bleeding from high risk varices showed that NSBBs alone or in combination with ligation achieved a better survival than ligation alone in patients with compensated cirrhosis, but not in patients with decompensated cirrhosis (121).

### Prevention of rebleeding

The very first meta-analysis (130) on the value of the use of NSBBs in recurrent bleeding prophylaxis of variceal hemorrhage already showed that NSBBs as compared to no active intervention not only significantly decreased the risk of bleeding during the observation period of about 2 years (mean improvement 20%), but also prolonged survival to a minor degree (increase in survival rate during a follow-up of around 2 years from 67 to 75%). There was no difference in mortality between ligation alone and ligation plus NSBBs for prevention of rebleeding. It is difficult to deduce from a recent network meta-analysis (3,526 participants, 48 randomized trials) the effect of NSBBs on survival when compared to other active treatments (131). It does not appear that there is a major difference. Interestingly, the potential beneficial effect on survival might be independent of hemorrhage protection (132).

A beneficial effect on mortality in the situation of rebleeding prophylaxis has been shown to be predominantly limited to patients in whom NSBBs (propranolol or carvedilol) reaches a drop in HVPG of at least 10% (133–135). In this respect it is noteworthy that insertion of a covered TIPS, which achieves the most effective drop of portal pressure has a higher impact on survival than standard of care (ligation and NSBBs). However, this was mainly due to TIPS placement in early temporal relationship to bleeding (125), while elective TIPS does not improve survival compared to drugs (76, 123). It remains an open question in this setting, whether hemodynamic non-responders (HVPG) profit from continuation of NSBBs treatment with respect to survival (136).

## NSBBs in cirrhosis: Controversies

### NSBBs in decompensated liver cirrhosis with ascites

It has been shown that early administration of NSBBs can prevent ascites formation in some patients with compensated cirrhosis (118, 121, 137). But the question was raised by the Paris working group whether it is useful to give NSBBs to patients with refractory ascites (138). In a prospective case-only study, they found that patients receiving propranolol had a significantly shorter survival as compared to those without NSBBs. The vast majority of patients without NSBBs–otherwise comparable–had no esophageal varices in this study, a fact that later became a matter of debate. There is now a number of reviews that carefully analyze the existing literature, as to whether NSBBs are appropriate in severely decompensated liver cirrhosis (14, 139). Some evidence suggests that ß-blockers can/should also be given in patients with ascites and decompensated liver cirrhosis (140–142) under strict control of pulse, blood pressure and renal function using an adjusted lower dosage (143). Further reduction of the cardiac index (CI) in the presence of primarily already reduced cardiac function in the sense of cirrhotic cardiomyopathy is certainly unfavorable (144, 145). Systolic blood pressure <90 mmHg, elevated creatinine levels above >1.5 mg/dl (better > 1.3 mg/dl?) or an increase in creatinine value are contraindications to starting or continuing the administration of NSBBs (14). Some authors regard application of carvedilol with its more pronounced effect on visceral and systemic hemodynamics as being contraindicated in patients with marked ascites (146). In any case, NSBBs should be dosed carefully in patients with reduced CI.

### Contraindications, side effects, duration of therapy, and adherence

In our own experience (76), nearly 10% of patients with cirrhosis had contraindications to NSBBs (such as refractory ascites, non-compliance, hepatic vein thrombosis, severe heart failure, or HRS type 1). In another randomized trial (147), 5% of eligible patients with liver cirrhosis had contraindications for propranolol. Complaints such as symptomatic hypotension, dizziness, impotence, and Raynaud symptoms occurred in nearly 70% of patients receiving propranolol, requiring withdrawal in 16% of the patients (of these 80% hypotension). In a controlled trial on early treatment of liver cirrhosis with NSBBs, 5 % of the screened patients had contraindications against NSBBs. Eight percent of patients discontinued NSBBs for side effects, as did 6% of the placebo patients (118). In another controlled trial on pre-primary variceal bleeding prophylaxis 18% of the patients had serious adverse events probably related to study medication (placebo 6%) such as bradycardia, fatigue, wheezing, claudication, and impotence (117). In a meta-analysis of eight RCT comprising 311 NSBB-patients in the setting of prophylaxis of first bleeding, side effects of NSBBs (mainly hypotension and breathlessness) required stopping treatment in 15% of the patients (148).

There is evidence that discontinuation of NSBB in patients with cirrhosis is associated with a high risk of rebleeding and that these patients may even have an increased mortality (147, 149, 150). Therefore, patients must be carefully selected for NSBBs, since it is aimed as life-long therapy.

Long-term drug application also concerns the assessment of compliance and adherence. Poor medication adherence is an important cause of inadequate treatment of long-term diseases (151). It is believed that 30–70% of hospital admissions in the US are due to non-adherence (152). The proportion of non-adherence to medications against hypertension, dyslipidemia, or diabetes is around 30% in the US (153).

With respect to liver disease, in one study, 23% of patients showed poor adherence to NSBB intake for bleeding prophylaxis from esophageal varices (154). Other small intervention studies showed that 25% of patients with cirrhosis have poor medication adherence and just under 50% showed good adherence (155). Under the conditions of a randomized trial 9% were non-compliant within a period of 2 years (147). Analysis of a large US database found around 60% of patients with variceal bleeding and decompensated cirrhosis receiving NSBBs. Of those, only 8% showed consistent use (156). Targeted care to improve medication adherence can reduce the rate of emergency hospital admissions in cirrhotic patients (157, 158).

### Prognostic markers and surrogates for endpoints

In the 1980s, it was shown that certain endoscopically definable criteria of varices (e.g., size or the so-called red color sign) are associated with higher blood pressure in the varices and their risk of bleeding (159–161). These endoscopic appearances have been used for decades to select patients, especially in primary prophylaxis of variceal hemorrhage with NSBB (162). As early as the 1960s, the Child classification was introduced and more or less modified over the years (163). Parameters from the Child classification were then combined with renal function (164). These systems are–with modifications–undisputed for the prediction of survival and also for selection for liver transplantation (128, 165). Their prognostic accuracy can be slightly improved by adding inflammatory parameters (166–168). To what extent these scores should be brought into the decision process for application of NSBBs is still in debate.

It is to the credit of Lebrec and his group (169), and later mainly Spanish and also Austrian working groups, to have introduced in a very consistent and careful way over the years the role of portal hypertension, measured as HVPG, for the prognosis of patients with liver cirrhosis, supported by many clinical studies (170, 171). Patients with an HVPG below 10 mmHg have a low risk of developing hepatic decompensation or death at least during the following 3–5 years. Patients with a HVPG > 20 mmHg have not only a high risk of early recurrent hemorrhage in case of variceal bleeding, but also a high risk of death (134, 172, 173). They benefit from the early enrollment for a TIPS in case of bleeding (173, 174). The decrease of HVPG > 20 % or to a value below 12 mm Hg, is a good criterion for protection against variceal hemorrhage, even ascites, and possibly for better survival (133, 134, 172, 175–178). According to some of these studies, the drop in HVPG of 10% is sufficient for the prognosis of prolonged survival. However, the value of this hemodynamic parameter as a surrogate for clinical end points in trials remains controversial. Among other reasons, because the measurement of HVPG is only performed by a limited number of centers in routine practice and because it is unclear how exactly other groups can measure HVPG (variability of measurement, but also intraindividual variability). More and more the measurement of liver or spleen stiffness is used for evaluation of portal hypertension (179), but its value to measure the response of portal pressure to NSBBs or to define clear thresholds is probably not sufficient.

Determination of pharmacokinetic parameters, including change of stereoselective metabolism in liver cirrhosis (180), are probably not of prognostic value for hemodynamic response to NSBBs. Studies on pharmacogenetics with respect to ß-AR gene polymorphisms and the action of NSBBs are sparse and inconclusive for patients with liver cirrhosis (181, 182).

## Epilogue: Pragmatism or perfection?

Although NSBBs have been used for the prophylaxis of variceal bleeding for four decades, a number of questions remain unanswered, as we have explained above. This concerns the choice of NSSB or the question of whether and how the expression of ß-AR in the different organs changes in liver cirrhosis. It remains also completely unexplored, how ß-AR-dependent intracellular signaling cascades change in a cell-specific manner with decreasing liver function. While we have focused a lot on the cardiovascular system in terms of NSBBs, we know very little about how they act in the diseased liver, especially with respect to liver resistance to portal flow. It is unclear to what extent biased signaling *via* ß-arrestin, in the event of carvedilol administration, exerts on the liver or on the heart in patients with liver cirrhosis. NSBBs may also have a double-edged effect on the immune system in liver cirrhosis. The question as to whether it is best to use NSBBs in a HVPG response-controlled manner remains open, and we do not know, whether patients who do not respond adequately with a drop in HVPG, will benefit at all from further administration of NSBBs. Also, we do not know to any great extent how the degree of liver cirrhosis or a change in albumin metabolism influence the effect of NSBBs. Last but not least, the standards for the optimal dose range of NSBBs in liver cirrhosis are also under discussion. Thus, there is much no man’s land in questions about pharmacokinetics and pharmacodynamics and the use of NSBBs in patients with liver cirrhosis.

Are NSBBs a good long-term therapy in routine clinical practice, given that about 10% of patients have primary contraindications to NSBBs, almost 20% have to discontinue NSBBs because of side effects, and given that no more than 50% of this patient group shows adequate drug adherence, although lifelong therapy is necessary? This issue is relevant, considering that in Western countries, most patients now have metabolic cirrhosis (food and/or alcohol). One wonders whether these patients are really compliant for such a therapy. That may be an unfair assumption. Sufficient data is lacking in this regard. There is also insufficient data on quality of life under continuous treatment with NSBBs in patients with liver cirrhosis. More studies on combination therapy–e.g., NSBBs with statins, angiotensin II receptor blockers, phosphordiesterase-5-inhibitors–drugs that might work against chronic inflammation in cirrhosis, or even the combination of NSBBs with a narrow-lumen TIPS are also necessary.

Can we answer these open questions with rigor, through more perfection? By individualizing the choice of NSBB or combined treatment? By regular monitoring of the HVPG? By better informing the patient and controlling drug adherence? By assessing the quality of life of the patients (there are hardly any studies on this)? Certainly not immediately. But some of these questions are worth further clinical research to achieve more perfection in the treatment of patients with liver cirrhosis using NSBBs.

**FIGURE 3:**
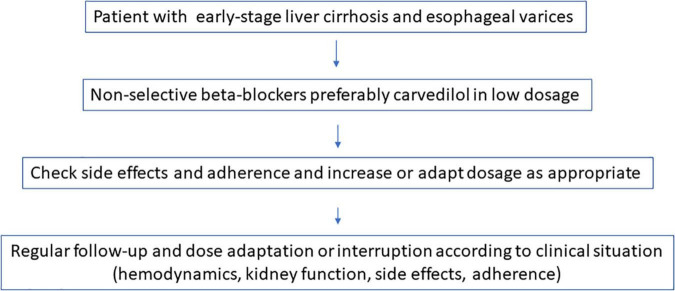
Non-selective ß-blocker (NSBB) in liver cirrhosis: The pragmatic approach.

On the other hand, clinical action requires pragmatism, taking into account the evidence, based on the available studies present. Controlled trials (RCTs) provide the best unbiased information about the effect of an intervention in medicine. And there are a lot of RCTs with respect to NSBBs and liver cirrhosis. For the single patient, RCTs show the best possible choice of intervention, but they will never give the answer as to how the individual will respond. Under these circumstances it might be easiest to start NSBBs–preferably carvedilol–very early and at a low dose so that the patient complies with the therapy, with attention to pulse reduction, monitoring renal function and blood pressure at regular intervals, and to choose an alternative therapy in case of intolerance or lack of adherence or deterioration of kidney function (Figure 3). All this must be done in consideration of the other medications the patient needs. This pragmatism should, however, be accompanied by further research, which demands perfection. To this end we suggest further studies (Table 2).

**TABLE 2:** Proposals for further research–non-selective ß-blocker (NSBBs) and liver cirrhosis.

**With respect to pharmacokinetics**
- Distribution, metabolism, excretion in decompensated cirrhosis
**With respect to pharmacodynamics**
- Signaling at different organs and cells, dependent on etiology and stage of liver cirrhosis
- Pleiotropic effects on intestinal and immune system
- Modulation by genetic background
- Biased signaling effects of carvedilol on intrahepatic resistance and fibrosis (increase/decrease?)
**With respect to individual behavior**
- Adherence to NSBBs
**With respect to concomitant drugs or interventions** **(TIPS, ligation)**
- Additive, complementary, or neutralizing effects
**With respect to dosage**
- Optimal tradeoff between side effects and efficacy
**With respect to selection of biomarkers under** **NSBBs–prediction of:**
- Bleeding
- Ascites
- HCC
- Survival

## Author contributions

TS wrote the first draft of the manuscript. MH, JT, and RS corrected and reformulated sections of the manuscript. RS supported editing the references. All authors contributed to the article and approved the submitted version.
